# Reshaping the Management of Allergic Rhinitis in Primary Care: Lessons from the COVID-19 Pandemic

**DOI:** 10.3390/ijerph192013632

**Published:** 2022-10-20

**Authors:** Baharudin Abdullah, Kornkiat Snidvongs, Niken Lestari Poerbonegoro, Budi Sutikno

**Affiliations:** 1 Department of Otorhinolaryngology—Head and Neck Surgery, School of Medical Sciences, Universiti Sains Malaysia, Kubang Kerian 16150, Kelantan, Malaysia; 2 Department of Otolaryngology, Faculty of Medicine, Chulalongkorn University, Bangkok 10330, Thailand; 3 Faculty of Medicine, Universitas Indonesia, Jl. Salemba Raya No. 6, Jakarta 10430, Indonesia; 4 Faculty of Medicine, Universitas Airlangga, Jl. Mayjen. Prof. Dr. Moestopo No. 6-8, Surabaya 60286, Indonesia

**Keywords:** allergic rhinitis, COVID-19, health literacy, primary care, self-care, telehealth

## Abstract

The COVID-19 pandemic presented unique challenges to the delivery of healthcare for patients with allergic rhinitis (AR) following its disruption and impact on the healthcare system with profound implications. Reliance on self-care for AR symptom management was substantial during the pandemic with many patients encouraged to only seek in-person medical care when necessary. The advantage of digital technology becomes apparent when patients and healthcare providers had to change and adapt their method of interaction from the regular physical face-to-face consultation to telehealth and mobile health in the provision of care. Despite the pandemic and the ever-evolving post pandemic situation, optimal management of AR remains paramount for both patients and healthcare professionals. A reshaping of the delivery of care is essential to accomplish this goal. In this paper, we present what we have learned about AR management during the COVID-19 pandemic, the role of digital technology in revolutionizing AR healthcare, screening assessment in the identification and differentiation of common upper respiratory conditions, and a framework to facilitate the management of AR in primary care.

## 1. Background

Allergic rhinitis (AR) is a common disease affecting up to 40% of the general population worldwide. In the Coronavirus 2019 (COVID-19) pandemic era, many observational studies analyzing the effect of asthma and chronic obstructive pulmonary disease on the risk of developing COVID-19 were conducted, while data on AR are limited. The risk of developing SARS-CoV-2 infection carried by AR patients, the outcomes of those with COVID-19 disease, and the COVID-19 influence on the allergic and nasal symptoms and the psychological status of AR patients, in both adult and pediatric populations, should be discussed. It seems that being an AR patient does not increase the risk of poor COVID-19 prognoses. The clinical manifestation of AR can be distinguished by COVID-19 symptoms. Treating AR adequately is also strongly recommended, especially during the pandemic. The COVID-19 pandemic has reshaped the delivery of healthcare, particularly for those with upper respiratory illnesses and chronic conditions, including AR. At present, COVID-19 is still a concerning global health issue. From our experience going through the critical phase of the pandemic, we can recall some of the challenges it has posed and put to use what we have learned to AR management in primary care. In this paper, we discuss these lessons and present a practical framework for management of AR in this new normal and beyond.

The management of AR is dynamic, and the pandemic has added unique challenges to this landscape. Distinguishing symptoms of AR from COVID-19 and other viral infections of the upper respiratory tract, such as the common cold and influenza, poses an increasingly significant concern. Limited access to physical and in-person healthcare saw many patients with AR rely on self-care strategies—such as self-diagnosis, over the counter (OTC) medication use, or even alternative complementary therapy—to manage their condition. Where medical consultations were necessary, patients and healthcare providers had to use digital technologies, such as telehealth and mobile health applications, to communicate beyond the physical primary care setting. Contrariwise, the emergence of an information epidemic caused the general public to be overwhelmed with health information and communication, which made it tricky for them to source reliable information on managing their health.

## 2. Navigating the Way out of an ‘Infodemic’

The health consequences of COVID-19 are widely known and discussed, both in contemporary literature and in society at large. This is the first pandemic in history where technology and social media have been used on a huge scale to keep people informed and connected. However, the technology people have relied on during the pandemic to provide safety could potentially also be harmful [1]. Termed ‘infodemic’ by the World Health Organization, this overabundance of information—some accurate and some not—has made it challenging for people to find trustworthy sources of information and guidance [2]. Studies have shown that misleading information spreads rapidly when there is distrust in the authorized information sources, or when credible information is difficult to verify [3]. Misinformation surrounding COVID-19 has posed significant challenges to primary care. Symptoms overlapping with AR, COVID-19, and other respiratory viruses can be baffling for patients, which prompted many to delay seeking medical advice or help [4,5,6]. The pandemic has exemplified the importance of health literacy and its impact on health and medical management [7].

## 3. The Role of Self-Care in Allergic Rhinitis

Allergic rhinitis carries a great burden, as a high percentage of patients have insufficient symptom control and their disease significantly impacts on their quality of life [8,9]. Patients with AR often underestimate the severity of their symptoms and tend to trivialize their disease [9]. This disconnect is problematic for both patients and healthcare professionals alike [10].

Primary care providers are an important source of health information for patients, as many patients choose treatment related to their health based on a recommendation from a trusted source, such as general practitioners (GPs) or pharmacists. Furthermore, primary care providers play a key role in educating their patients on preventing diseases and treating simple ailments at home [11,12]. Given the non-critical nature of AR, most patients were encouraged to self-monitor during the COVID-19 pandemic and only seek in-person medical care when necessary. As a result, the reliance on self-care for AR symptom management was substantial [9].

The term self-care is not exactly commonplace in parts of Southeast Asia. However, it is widely practiced in many countries throughout the world. Many people regularly self-diagnose minor illnesses and access household remedies and OTC medicines prior to consultation with a physician [12]. For AR, these include treatments that can be procured at a pharmacy, such as oral H1-antihistamines. Access to self-care medicines is valuable to lower the burden on primary care and reduce the potential of developing chronic health conditions by initiating early treatment [11]. A study of 18 million adults with AR in the United States found that consumers saved up USD 90 million annually when half of them avoided one visit to the doctor yearly by using self-care therapy [11].

However, it should be emphasized that self-care produces the greatest value only when patients have a high degree of health literacy, understand the value of preventive care, and are confident and empowered to make their own health decisions [12]. There is evidence pointing to lower health literacy being associated with negative self-management outcomes, including low medication adherence rates and poor patient–provider communication and patient knowledge [13,14,15,16,17,18,19]. Thus, patients should be encouraged by primary care providers to boost health literacy and play an active role in their health [11,12].

## 4. Advancing Self-Care through Technology

Communication between healthcare providers and their patients is fundamental to patient care and could not be replaced by digital technology [20,21]. Nevertheless, the role of technology in driving self-care has become increasingly evident throughout the COVID-19 pandemic, especially for a chronic condition such as AR.

### 4.1. Telehealth and mHealth: Keeping the Lines of Communication Open

COVID-19 has reshaped how conventional healthcare is delivered, including how patients and healthcare professionals interact [4,22]. Combining the need for regular consultations with the protection of healthcare workers and patients was a significant challenge during the pandemic [23].

Synchronous interactions that occur in real time between a patient and healthcare provider either via audio or video call are now frequently relied upon to supersede face-to-face visits [24,25]. A survey had found telemedicine to be an essential part of practice for approximately 43% of healthcare professionals [26].

Telehealth in allergy cases has also been linked with high patient satisfaction [25,27]. A study of telehealth encounters during the pandemic revealed 75% of patients felt their consultation was as satisfactory as an in-person visit [25]. Another research demonstrated a higher satisfaction rate of 98.8% for telehealth consultation [27]. Moreover, patients’ satisfaction using telehealth was applicable for both new and follow-up patients [27].

A good example of telehealth is the mobile health technology or mHealth apps. mHealth apps allow healthcare providers and patients to remain connected, offering the possibility of high-quality consultation outside of the customary office hours. In addition to enhancing patient–physician communication, their utilization reduces use of healthcare resources and economic costs [28,29]. The MASK app is one of the highest ranked mHealth apps available from the Apple App Store and Google Play Store in selected countries such as Europe or Australia, whereby further details will be described in the subsequent section. Apart from fulfilling the required criteria of easy accessibility, user-friendly, and compatible with the self-management of AR principles, users of the MASK app reported it to be engaging, highly intuitive to use, highly visually appealing, and provides high-quality information [9].

There are certain situations where telehealth may not be appropriate for patients or healthcare providers. In determining telehealth as an option to the long established in-person visit, patients must have the cognizance of its role in the delivery of care. In order to make this selection, patients must have a good level of health literacy and an understanding of the advantages and disadvantages in each and every circumstance.

Notwithstanding this prerequisite, the advantages of telehealth for AR are clear where its application is practical, potentially more cost effective and less time consuming. It also minimizes the risk of exposure to healthcare providers and patients from contracting viral respiratory illnesses. In recent times, telehealth has been shown as a useful tool to effectively manage AR [23,27,30]. The key considerations for the use of telehealth in patients with AR are outlined in Table 1.

### 4.2. Patient Empowerment through Mobile Technology

Digital technologies have been acknowledged as a significant tool by the World Health Organization in improving the health systems in countries to achieve the health-related Sustainable Development Goals that include universal health coverage [31]. At the beginning, mobile health technology was thought mainly pertinent in developed countries. With increasing competition among the smartphone manufacturers, smartphones become much more affordable. This leads to rapid growth of smartphone ownership around the world. Among the mobile telephone subscriptions across the world, the majority were found in less developed regions comprising low to middle income countries [32]. This allows a transformation from a traditional healthcare delivery towards a more efficient and integrated method designed to form a cost-effective and patient-oriented management.

Among the benefits of mobile technology are self-monitoring with an electronic diary, targeted feedback, and personalized education of individual patient. Their utilization could enable and augment self-management of patients in their daily lives. A review of related randomized controlled trials revealed that text messaging via mobile phone improves the rate of medication adherence in AR patients [33]. This approach offers a cost-effective and practical option for patients particularly in low- and middle-income countries where lack of transport and high cost represent major concerns.

By personalizing the treatment according to the condition and need of patients, mobile technology helps to improve treatment adherence. For instance, the daily uptake of medication can be monitored through the smartphone video camera system for verification by the health providers directly or by recording. By using this monitoring system, any issues in complying with the given treatment can be detected, to allow counseling of patients and adjustment to the treatment to be made accordingly.

### 4.3. Patient Journey in the Digital Age

The shift of a patient journey from the traditional physician-centric approach to a digital patient-oriented method has been proposed [34]. The shortcomings of the traditional method have been highlighted. There was the mistaken belief of using simple logic to epitomize patient behavior, which erroneously omits the subjective element in the decision-making process of each patient, together with overlooking the emotional and behavioral features related to the diagnosis and treatment process. The traditional method also did not take account essential events outside a physician’s clinic, such as prescription fulfilment and cost burden. The digital patient journey captures the emotional, informational, and behavioral needs of a patient in a patient-centric approach. The comprehensive integrated and connected services by digital technology allow better communication and create efficiencies across the range of care.

The digital patient journey addresses the complex behavioral, emotional, clinical, and informational needs of patients. A patient’s behavior is fundamental in implementing a successful treatment’s goal. Factors like emotions, surroundings, and choice of treatment impact a patient’s behavior, for example, the belief that a treatment would be ineffective affects adherence. A physician showing empathy can bolster a patient’s trust and lead to improved communication. When the patients feel that they are understood and well acknowledged, they will be more receptive towards the physician’s counsel. The delay in confirming a diagnosis and late delivery of care to patients must be avoided in addressing their clinical needs as they may cause unnecessary physical and mental stresses for the patients. The informational needs are critical to ensure that patients receive high quality care when essential information can be communicated swiftly along their digital journey.

In real world practice, patients not only suffer from AR but also other comorbid conditions. This journey is well illustrated by the use of an app to manage AR with comorbid conditions. Multimorbidity in allergic airway diseases is well established [35]. The multimorbidity impacts the daily fluctuating symptoms, contributes to the severity and impairs work and activities. An information and communication technology system centered around the patient such as the MASK app is available in the majority of European countries with a growing number of different languages [36,37,38]. It contains all medications adapted to each country together with visual analogue scales to provide assessment of control and treatment response. MASK is scaled by the EU European Innovation Partnership on Active and Healthy Ageing strategy with support from EU grants and the World Health Organization [39,40]. The use of MASK has enabled recognition of day-to-day intraindividual variability in patients with allergic multimorbidity. The differentiation of patients having only AR from those having rhinoconjunctivitis in studies using MASK provides significant proofs in their management. The studies disclosed that patients with allergic multimorbidities were at risk of a greater severity of allergic diseases [41,42,43]. Among the notable findings were ocular symptoms more common in polysensitized patients regardless of the asthma status and severity of nasal symptoms was directly related to ocular symptoms. Hence, ocular symptoms must be assessed in all patients with polysensitized allergy and managed accordingly.

### 4.4. Harms and Limitations of Telehealth

The availability of a large number of mHealth apps focusing on a variety of concerns of the general public can create confusion. Even though many patients rely on the apps in their everyday lives, a large number of the apps have not been validated or approved by the regulatory institution despite being used widely by patients [28]. This brings into question their effectiveness and reliability, which could lead to potentially harmful consequences.

Potential weaknesses become apparent related to the diagnosis and treatment by mHealth apps [44,45]. The reporting of their symptoms and response to treatment by patients could be misleading as this is dependent on their informatics literacy rate. Patients tend to express the symptoms that bother them the most and suppress others which they think are insignificant. Such reporting portrays a vague pattern that may create difficulty for physicians to interpret and grasp the nature and characteristics of each patient. Moreover, not all patients might be receptive towards the use of an electronic diary. Though electronic diaries have been proven to be more reliable than paper diaries [46], its accomplishment is still subject to patients’ acceptance.

There are also specific concerns surrounding its use, including medico-legal, security and privacy implications, confusion or uncertainty around billing and reimbursements, and technical obstacles arising from the difficulty and reliability of a virtual physical examination or self-examination by patients, especially related to the ear, nose, and throat. Further improvements to the program are warranted to ensure reliable diagnosis and sufficient control of AR symptoms can be secured.

### 4.5. Telehealth for Special and Vulnerable Populations

Special and vulnerable populations include children, elderly, or socioeconomically disadvantaged people. Members of vulnerable populations often have health conditions that are exacerbated by unnecessarily inadequate healthcare [47].

The phenomenon of an aging population is affecting most countries, with an increasing burden on the healthcare systems. The majority of older adults are living with at least two chronic conditions [48]. Older adults are a diverse population. Some are still actively working or are family caretakers, while others need an advanced degree of care. Generally, the elderlies have a positive attitude towards the rapid and diverse development of the surrounding digital environment [49]. However, older adults may have minimal experience and familiarity with technology, and their chronic condition may also affect the usability of digital technology for self-care. It has emerged that the main difficulties of the elderly to use digital technology were due to vision and hearing issues [50].

In order to encourage self-care via digital means in the elderly, the user interface needs to be simple, easy to use, and provide consequential interaction and feedback [51]. Devices that allow patients to follow written instruction and modify care based on biological information need to be efficient and user-friendly. In providing a telehealth service, accessible instructions, for example, the use of illustrations, along with other information, are essential. For elderly in the care of others, early involvement of caregivers including family members, social workers, or community health workers is crucial.

The application of telehealth in children requires trust and commitment from the parents. Strategies need to be adopted to advance telehealth for such a purpose [52]. Awareness of telehealth must be propagated as an alternative option to face-to-face consultation. This must be followed through with strategies to build interest and knowledge about telehealth to engage in telehealth visits when the need arises. Thereupon, there needs to be an increase in the utilization of telehealth to successfully provide care. It goes without saying that continuous engagement with the parents to address their concerns and worries will go a long way to gain confidence and acceptance of telehealth.

The obstacle of limited internet access in socioeconomically disadvantaged people or those living in rural communities can be overcome by using shared devices or networks in the utilization of telehealth [53]. Nonetheless, there needs to be a balance between privacy and maximizing access. Hence, patients must also be given tips on the measures to safeguard their personal information.

## 5. Managing Allergic Rhinitis in Primary Care

Self-management—including symptom monitoring, allergen avoidance and adhering to a treatment plan—is the mainstay of management for AR in the real world. However, due to the dynamic nature of AR management, interaction with healthcare providers is essential, particularly GPs and pharmacists [9,10]. Recognition of the disease, appropriate medication selection, exact treatment management, review of the effectiveness of medications, and offering counseling and referral where needed are some of the care pathways requiring interaction with primary care providers [54,55]. Despite the use of medications, patients with AR typically present to primary care with symptoms that are not well-controlled [56]. Real-life observational studies have shown that many patients did not seek advice from physicians and self-medicate to treat their symptoms by using OTC medications [56].

Allergic Rhinitis and its Impact on Asthma (ARIA) guidelines provide evidence-based recommendations for the management of AR and asthma and adherence to such guidelines has been shown to lead to better patient outcomes [55]. Despite the importance of these guidelines, the literature suggests that there is a low level of awareness at the primary care level and that guidelines may not always reflect patients’ needs or real-life experiences [55,57]. Furthermore, patient care cannot be achieved using guidelines and treatment algorithms alone. Understanding the patient profiles and selecting the appropriate treatment strategy based on factors such as age, symptoms, severity, patient preferences, cost, and individual circumstances is the key to optimal AR management [57,58].

### Screening for Allergic Rhinitis and Other Respiratory Diseases

The COVID-19 pandemic has imposed significant challenges on primary care, where factors such as the need to protect oneself from exposure and contracting infection from others and the obligation to maintain a standard of healthcare obfuscated the handling and management of upper respiratory disorders [57,59]. As COVID-19 joins the long list of other respiratory conditions seen in the primary care setting, it is now more imperative than ever that pharmacists and GPs must be able to identify their symptoms and differentiate the upper respiratory disease [54,55]. Concurrently, appropriate guidance and treatment selection must be provided to help improve patient outcomes.

**Figure 1:**
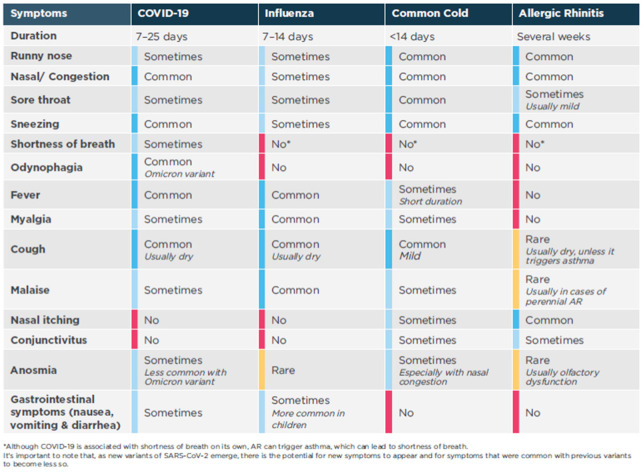
Identification and differentiation of common upper respiratory conditions.

The widespread availability and use of antigen rapid tests for COVID-19 has allowed the general public to distinguish it from other upper respiratory symptoms. Nonetheless, due to the nature of the common upper respiratory tract infections which share common symptoms such as nasal congestion, rhinorrhea, sore throat, and sneezing, it is arduous to accurately identify the type of ailment, or group of ailments, based on symptoms alone [59,60,61,62,63,64,65,66]. At the start of the pandemic, COVID-19 commonly presented as a flu-like illness with fever and persistent cough as its main symptoms. The presentation gradually shifted to milder symptoms of runny nose, sore throat, nasal congestion, and aches and pains or diarrhea. Figure 1 highlights additional information on symptom differentiation for AR, COVID-19, and common respiratory viruses. To confirm the presence of AR, the patient’s symptoms should be corroborated and other conditions must be considered and excluded [59,60,61,62,63,64,65].

**Figure 2:**
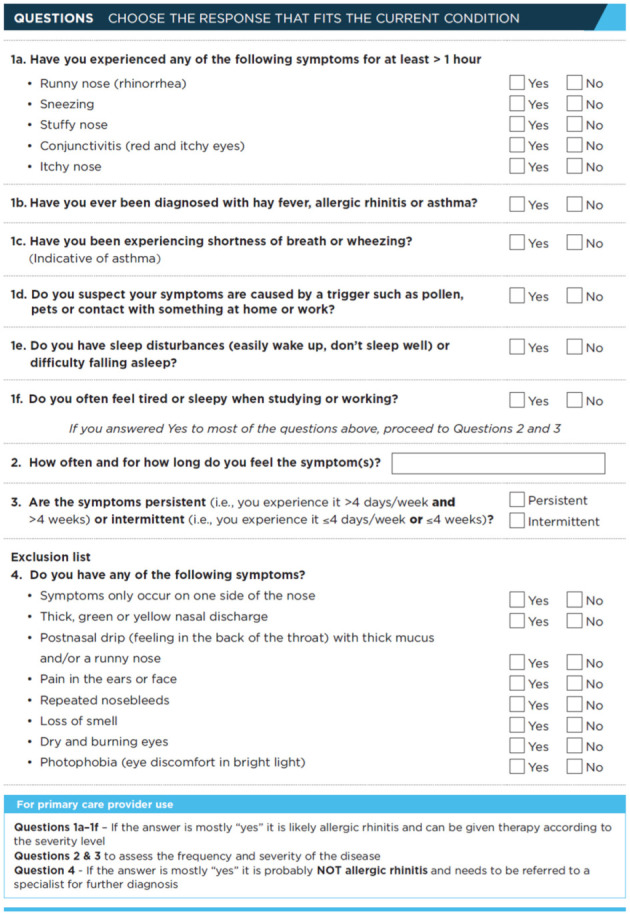
Screening patients for symptoms of allergic rhinitis.

When AR is suspected, using the following screening questionnaire in the primary care setting serves as an assessment tool to aid the diagnosis of AR and provide timely management advice according to patient responses (Figure 2). The tool is developed to help primary care providers accurately and efficiently manage AR in the community [62,67,68]. It can be used during a telehealth consultation if an in-person visit is not feasible or preferred.

The first three questions (Q1–Q3) from the questionnaire in Figure 2 help to characterize, classify, and identify AR in addition to the assessment of its effect on patients. The last fourth question (Q4), which is equally important, helps to exclude the presence of other nasal conditions oblivious to the patients. A survey among health professionals from different specialties by ARIA revealed a consensus that symptoms of sneezing, nasal obstruction, nasal itchiness, rhinorrhea, and ocular itchiness are the main presentation of AR [63]. The overwhelming majority strongly agree that patients with three or more nasal symptoms at presentation are distinctive characteristics of AR, which can be used to distinguish it from COVID-19 infection.

Persistent unilateral nasal obstruction (“symptoms only occur on one side of the nose”) signifies a structural nasal septal deviation which can simulate AR symptoms or aggravate a concomitant AR condition. Nasal septal deviation requires further referral to an otorhinolayngologist, where surgery can be considered to correct the structural abnormality of the septal framework. In patients who have nasal septal deviation with concurrent AR, surgery will also improve the delivery of intranasal sprays postoperatively [69].

When patients presented with an early symptom of smell loss without nasal obstruction, the key consideration is COVID-19 infection [70]. Early smell loss is a characteristic pathognomonic feature indicating spread of the virus along the olfactory nerve upon gaining entry into the nose. The absence of fever, cough, or sore throat does not preclude this infection and the use of a screening test for COVID-19 is advisable. The sense of smell usually recovers spontaneously within 6 weeks, though in certain cases it could be longer.

The presence of a mucopurulent thick white, yellowish, and greenish nasal discharge together with the additional symptoms of nasal obstruction, loss of smell, postnasal drip, and facial pain are highly indicative of chronic rhinosinusitis, which is a sinonasal inflammatory condition [71]. It must be reiterated that the presence of an allergy does not necessarily exclude chronic rhinosinusitis. An allergic phenotype of chronic rhinosinusitis has been well described [72] and must be duly distinguished from AR by its characteristic aforementioned symptoms supported by nasoendoscopic examination. Further investigation by imaging using either computerized tomography or magnetic resonance imaging of the sinuses maybe required [73].

Pain in the face or ear with photophobia and dry or burning pain of the eyes are features of vascular, neurogenic, or autoimmune conditions such as migraine, trigeminal neuralgia, Sjogren’s, etc. [74,75,76]. A referral to a neurologist or rheumatologist is recommended for further investigation and confirmation.

The symptom complex of nasal obstruction, repeated nose bleeds, and loss of smell might herald a sinister condition such as sinonasal tumors [77,78]. The presentation of sinonasal tumors at an early stage is difficult to be discriminated from AR or chronic rhinosinusitis. It is not uncommon for a sinonasal squamous cell carcinoma or olfactory neuroblastoma to be initially treated as an inflammatory condition prior to the final diagnosis by experts upon poor response to pharmacotherapy [79]. A high index of suspicion is paramount for prompt referral for specialist care to commence, as early treatment is associated with a superior prognosis and outcome [80].

## 6. Framework for the Management of Allergic Rhinitis

**Figure 3:**
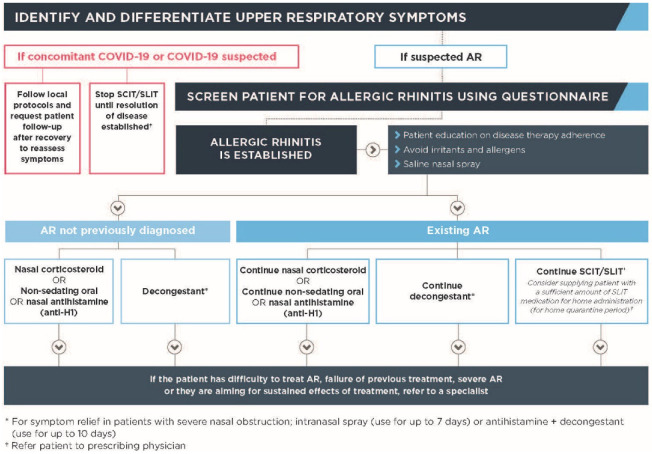
A framework for managing allergic rhinitis in primary care.

The following framework has been developed using current global resources from the Asthma and Allergy Foundation America, Center for Disease Control and Prevention, European Academy of Allergy and Clinical Immunology, European Forum for Research and Education in Allergy and Airway Diseases, and the World Health Organization, among other sources (Figure 3) [4,56,59,60,61,63]. Management guidance is based on previous assessment and according to confirmation of AR diagnosis—previously diagnosed with AR or suspected to have AR.

A consensus among the experts stated that AR management should be maintained during the COVID-19 pandemic, including allergen avoidance measures, patient education, pharmacotherapy, and allergy immunotherapy, particularly when COVID-19 is not confirmed or suspected [57]. Failure to do so could accelerate disease progression and lead to adverse health effects. Different patient profiles should be considered to guide the choice and use of medication in primary care [55].

Underpinning this framework are the principals of self-care. Patients should be encouraged to identify their symptoms and be prompted to discuss them with a primary care provider. This relies on the patient having a good level of health literacy and the ability to recognize the characteristics, severity, and impact of their symptoms. Once a patient has been assessed, education and self-management strategies can be delivered. When determining how communication and consultation can occur, consider the advantages and disadvantages of an in-person visit versus telehealth, noting that a number of methods may need to be used throughout the course of treatment. This framework and the other tools presented in this paper have been developed for primary care to screen and assess patients for AR in the absence of an in-person visit. Their implementation via telehealth and other digital means will provide a useful way to bridge the gaps in communication as digital health technology continues to advance.

This framework is appropriate for patients with AR that have not been previously diagnosed or have mild to moderate AR. Patients with a confirmed diagnosis of severe or difficult to treat AR or failure of previous treatment should be referred to specialist care as needed.

In patients who have never been diagnosed with AR, a thorough and comprehensive history is vital to establish the diagnosis and differentiate the many types of rhinitis [25]. Symptoms triggered on exposure to either indoor or outdoor allergens are highly suggestive of AR. Family history of atopic conditions, such as bronchial asthma and atopic dermatitis, or other siblings with AR are pointers towards the possibility of AR. On the other hand, short generalized symptoms associated with occasional fever denotes viral infection or infectious rhinitis. With the use of a webcam or mobile camera, facial inspection could illustrate dark discoloration under the lower eyelids (allergic shiners), a transverse line above the nasal tip (allergic salute), and facial features of a mouth breather, indicative of chronic allergic condition [81]. Using the same method, conjunctivitis, which is a component of AR, could be visualized. Other suggestive clues are history of enlarged tonsils and postnasal drips implying chronic rhinitis.

Once the diagnosis of AR is established, the initiation of treatment by using OTC medications and counseling are similar to office-based practice. The options of therapy in self-care include most of the available useful classes of medicines, such as second-generation oral antihistamines and intranasal corticosteroids [82]. With knowledge and advice, most patients would be able to manage their symptoms adequately without ongoing medical supervision. Counseling which includes avoidance of allergens is facilitated further by the use of video camera system, allowing the physician to see directly into the patient’s home and provide supervision on trigger avoidance. When any patient is in need of further guidance, the use of this technology enables direct communication with a physician.

When first line treatment is ineffective or the diagnosis of AR is doubtful, a scheduled office visit to a physician should be planned for further investigation including a confirmatory allergy test (skin prick test or serum specific immunoglobulin E test) [25]. The identification of the allergens facilitates the discussion and counseling for the consideration of initiating immunotherapy. Along the same vein, AR patients with comorbid bronchial asthma and atopic eczema are evaluated meticulously, aimed at the comprehensive management of the allergic conditions.

In the customary face-to-face consultations, ARIA recommended a step-wise approach in initiating and choosing medications for AR patients [67]. Likewise, treatment regimens are tailored to individual patients based on nasal symptoms, severity, and associated atopic disorders in the self-care approach. To treat intermittent symptoms, second-generation oral antihistamines taken on a needed basis are appropriate. A daily use of intranasal corticosteroid spray or daily second-generation oral antihistamines is recommended for persistent symptoms. The choice of an intranasal corticosteroid nasal spray or an oral antihistamine depends on individual preferences with regard to comfort, cost, availability, effectiveness, and tolerability [83]. Using the same consideration, the option of initiating treatment with a topical antihistamine may be offered to patients who desire a faster and complete symptom resolution.

In the re-assessment of patients following treatment, a continuation of the medications is advised for those who responded to the initial therapy but still have residual symptoms. On the other hand, a step-up therapy is incumbent for patients that have showed poor or no response to first line treatment. The use of combined second-generation oral antihistamines and intranasal corticosteroid sprays is common practice in the community. However, combining them both has been shown to have no added benefit to using intranasal corticosteroid sprays alone [84]. Instead, the use of combined intranasal corticosteroids and intranasal antihistamines is recommended [83]. Though intranasal corticosteroids are the mainstay of treatment for allergic rhinitis, their peak effect may take several hours to days, with maximum effectiveness observed after two to four weeks of use [85]. In comparison, intranasal antihistamines have an onset of action within 15 min that lasts up several hours [86]. Their combined use has been demonstrated to control most AR symptoms and is ideal as an optimal therapy in AR patients [87]. Another option for combined therapy is the use of intranasal corticosteroids with leukotriene antagonists [87]. A leukotriene antagonist such as montelukast is shown to be beneficial, especially for bronchial asthma patients with night-time symptoms [88]. AR patients with concomitant bronchial asthma may benefit from this combination treatment. Failure of pharmacotherapy may entail the administration of immunotherapy [87]. At this point, patients should be counseled to schedule for a physical consultation to assess their suitability for immunotherapy.

For patients that have exhibited a reduction of symptoms and a stable condition, step-down advice is given. Treatment frequency is de-escalated from regular intake to a needed basis. Similarly, in patients on a combined treatment, treatment is scaled down to a monotherapy.

In general, initiation and continuation of treatment can be facilitated by the use of this framework without having to carry out high-risk additional measures such as rhinoscopy, nasal endoscopy, nasal provocation testing, and smell and taste testing with an in-person consultation, carried out only when necessary [23].

It must be pointed out that the control of AR symptoms plays a vital role in reducing transmission of infectious disease, primarily COVID-19. Symptoms of AR such as sneezing, rhinorrhea, and coughing arising from postnasal drips predispose to aerosol formation responsible for viral spread. It is also imperative for patients to adhere to the standard pharmacological treatment for AR and its comorbid conditions such as bronchial asthma to prevent exacerbation of diseases and development of subsequent complications. Though asthma is not a potent risk factor in the progression of the COVID-19 disease [59], viruses are known common triggers of asthma exacerbations, which can potentially complicate the management and recovery process [89].

Currently, in the post-pandemic state, people can easily go to clinics and may not need telehealth and self-care. Notwithstanding, it may still be advantageous for certain geographical areas. In general, healthcare resources in the low- and middle-income countries are insufficient, with the available ones unevenly distributed due to the rural–urban gap [90]. Various opportunities of telehealth showed its potential in strengthening the healthcare system in low- and middle-income countries [91].

Implementation of telehealth in developing countries helps to reduce the inequality gap by providing a more cost-effective healthcare service, early disease detection, increased accessibility of basic health education, and improving the management of diseases [92]. The barriers to adapting telehealth in developing countries include the paucity of a good internet connection to access the service and lack of readily available infrastructure to support such services [93]. Furthermore, due to the wide domination of the English language on the internet, users must have some proficiency of the language [94]. Taken together, these constraints make it problematic to commence and implement telehealth.

Despite these issues, it is becoming abundantly clear that people living in rural or hard-to-reach areas will benefit from a telehealth service, specifically having to fork out the additional cost for transportation [53].

## 7. Conclusions

Although the COVID-19 pandemic has changed the way healthcare is provided, adjustments from the usual delivery of care by the healthcare provider and patient can ensure a successful management of AR. As the world shifted through various phases of the pandemic, healthcare providers can continue to provide quality care by employing an ingenious approach and embracing the use of technology, while maintaining the fundamentals of AR management. With patients commonly presenting to primary care with upper respiratory conditions, it is imperative that primary care practitioners understand the importance of their role in caring for patients with AR. The way in which patients access healthcare will certainly evolve, but self-care and technology remain relevant as important tools in AR management. Steps that GPs and pharmacists can take to optimize AR management now, and beyond the COVID-19 pandemic, include:

1. Recognizing self-management as the foundation for AR management and promoting patient self-care through education and support;
2. Disseminating accurate health information and building trust among people regarding health information sources and services;
3. Empower patients to increase their health literacy and to feel confident engaging in self-care;
4. Keeping the lines of communication open, whether it be through in-person interactions or the use of technology, to provide safe and effective care;
5. Urging patients to use digital self-assessment tools and mobile health applications to bridge gaps in health communication and support the notion of self-care;
6. Screening patients who present with upper respiratory symptoms to identify the underlying cause and guide patients to the most appropriate treatments for their condition, with consideration for the patient’s individual circumstances;
7. Continuing AR treatment during the COVID-19 pandemic.

## Figures and Tables

**Table 1:** Key considerations for telehealth use in patients with AR.

1. Telehealth enables interaction between patients and healthcare providers in the absence of an in-person visit
2. In-person visits for those with high-care needs should be given a priority
3. Evaluate delivery of care to determine whether telehealth versus in-person is appropriate based on: Cost Time Expectations Specific diagnosis Likelihood for adherence with prescribed medication regimens Follow-up needs
4. Telehealth is conceivable for all patients with AR, either controlled or non-controlled
5. Face-to-face evaluation is opted for: patients with low COVID-19 risk patients who require further investigation or require initiating sublingual immunotherapy (SLIT) patients not responding to initial treatment (either the symptoms do not fully or partially resolve).
6. In the assessment of symptoms, questionnaires can be prefilled by patients prior to the telehealth appointment
7. Step-up medications can be advised and prescribed via telehealth in non-controlled patients

## Data Availability

Not applicable.
